# Dual-phase xenon time projection chambers for rare-event searches

**DOI:** 10.1098/rsta.2023.0083

**Published:** 2024-02-05

**Authors:** Laura Baudis

**Affiliations:** Physik-Institut, University of Zürich, Winterthurerstrasse 190, Zürich 8057, Switzerland

**Keywords:** dark matter direct detection, astrophysical neutrinos, neutrinoless double-beta decay

## Abstract

In the past decade, dual-phase xenon time projection chambers (Xe-TPCs) have emerged as some of the most powerful detectors in the fields of astroparticle physics and rare-event searches. Developed primarily towards the direct detection of dark matter particles, experiments presently operating deep underground have reached target masses at the multi-tonne scale, energy thresholds of approximately 1 keV and radioactivity-induced background rates similar to those from solar neutrinos. These unique properties, together with demonstrated stable operation over several years, allow for the exploration of new territory via high-sensitivity searches for a plethora of ultra-rare interactions. These include searches for particle dark matter, for second-order weak decays, and the observation of astrophysical neutrinos. We first review some properties of xenon as a radiation detection medium and the operation principles of dual-phase Xe-TPCs together with their energy calibration and resolution. We then discuss the status of currently running experiments and of proposed next-generation projects, describing some of the technological challenges. We end by looking at their sensitivity to dark matter candidates, to second-order weak decays and to solar and supernova neutrinos. Experiments based on dual-phase Xe-TPCs are difficult and, like all good experiments, they are constantly pushed to their limits. Together with many other endeavours in astroparticle physics and cosmology they will continue to push at the borders of the unknown, hopefully to reveal profound new knowledge about our cosmos.

This article is part of the theme issue ‘The particle-gravity frontier’.

## 1. Introduction

The idea of a time projection chamber (TPC), ‘born on a waning winter afternoon’ in February 1974 [1] was first realized and successfully applied in particle physics experiments at accelerators. In the last decades, the concept evolved and was tailored to a large range of applications, most notably to experiments in astroparticle and nuclear physics searching for rare interactions deep underground. Present and future experiments designed to observe interactions of dark matter and other exotic particles, second-order weak nuclear decays, as well as neutrinos from a variety sources are based on TPCs operated with pure noble elements, either in gaseous or liquid form. As in the original idea by Nygren [2], modern TPCs capture the x–y information of events, along with their drift time, allowing for a three-dimensional position reconstruction if the start time is known.

The two most common noble fluids employed as detector media in present TPCs for rare-event searches are argon and xenon. Here we will focus on dual-phase (liquid and gas) xenon TPCs (Xe-TPCs). For general reviews of experiments based on noble elements, we refer the reader to Aprile & Doke [3], Chepel & Araujo [4] and Gonzalez-Diaz *et al.* [5]. Historical introductions, the physics and technological details, as well as applications of noble gas and electron emission detectors are detailed in [6,7].

This article is structured as follows. In §2, the properties of xenon as a radiation detection medium will be briefly reviewed. The working principle of two-phase Xe-TPCs and their three-dimensional imaging capabilities are introduced in §3, while their energy calibration and resolution are discussed in §4. Section 5 looks at past, current and future detectors, and at some technological challenges. Sections 6 and 7 will discuss the search for dark matter and other exotic particles, and for second-order weak decays, respectively. Section 8 will scrutinize the potential of dual-phase Xe-TPCs to detect neutrinos from the Sun and from core-collapse supernovae, while §9 will conclude the brief review.

## 2. Properties of xenon as radiation detection medium

Xenon is an excellent scintillator and good ionizer in response to the passage of radiation. In a TPC, the simultaneous detection of ionization and scintillation allows for the identification of the primary particle interacting in the medium based on the linear energy transfer, dE/dx, and for the determination of the three-dimensional position of an interaction with sub-mm (in thez-coordinate) to mm (in the x–y-coordinate) precision. Table 1 lists some of the physical properties of xenon, relevant for practical aspects of a detector. The high atomic mass number and high liquid density allows for compact, large and homogeneous detector geometries with efficient self-shielding against external radiation, given that the cross-sections for the photoelectric effect, Compton scattering and pair production scale as $Z^{5}/E_{\gamma }^{7/2}$, $Z/E_{\gamma }$ and $Z^{2}\ln\left(2E_{\gamma }\right)$, respectively, for incoming X-rays and gammas with energy $E_{\gamma }$. The radioactive isotopes ${\;}^{124}\mathrm{Xe}$, ${\;}^{126}\mathrm{Xe}$, ${\;}^{134}\mathrm{Xe}$ and ${\;}^{136}\mathrm{Xe}$ have very long half-lives, and their second-order weak decay modes are subject to investigation, as we will show in §7.

**Table 1.** Physical properties, volume fraction in the atmosphere, radioactive isotopes of the noble element xenon.

property (unit)	Xe
atomic number	54
mean atomic mass ($g\;{\mathrm{mol}}^{-1}$)	131.29
boiling point $T_{b}$ at 1 atm (K)	165.0
melting point $T_{m}$ at 1 atm (K)	161.4
gas density at 1 atm and 298 K ($g\;l^{-1}$)	5.40
gas density at 1 atm and $T_{b}$ ($g\;l^{-1}$)	9.99
liquid density at $T_{b}$ ($g\;{\mathrm{cm}}^{-3}$)	2.94
volume ratio	526
dielectric constant of liquid	1.95
volume fraction in Earth’s atmosphere (ppm)	0.087
isotopes with spin, abundance (%)	${\;}^{129}\mathrm{Xe}$, 26.44; ${\;}^{131}\mathrm{Xe}$, 21.18
radioactive isotopes,	${\;}^{136}\mathrm{Xe}$, 8.87; $2.2\times 10^{21}$ [8,9]
abundance (%) and $T_{1/2}$ (y)	${\;}^{124}\mathrm{Xe}$, 0.095; $1.1\times 10^{22}$ [10,11]
	${\;}^{134}\mathrm{Xe}$, 10.4; $>8.7\times 10^{20}$ (90% C.L.) [12]
	${\;}^{126}\mathrm{Xe}$, 0.089; $>1.9\times 10^{22}$ (90% C.L.) [13]

In a xenon detector, the energy loss of an incident particle is shared between ionization, excitation and sub-excitation electrons liberated in the ionization process. The average energy loss in ionization is slightly larger than the ionization potential, as it includes multiple ionization processes. The energy $E_{0}$ transferred to the medium, for light particles such as electrons, is [14]

2.1 $$E_{0}=N_{i}E_{i}+N_{\mathrm{ex}}E_{\mathrm{ex}}+N_{i}\epsilon ,$$

where $N_{i}$ and $N_{\mathrm{ex}}$ are the mean number of ionized and excited atoms, $E_{i}$ and $E_{\mathrm{ex}}$ are the mean energies to ionize or excite the atoms and ϵ is the average kinetic energy of sub-excitation electrons, the energy of which goes into heat. For liquid xenon, the value of ϵ is in the range 4.65--5.25 eV. In its condensed phase, xenon exhibits a band structure of electronic states, and if we divide all terms in equation (2.1) by the band gap energy $E_{g}$ and use the $W_{i}$-value, defined as the energy required to produce one electron–ion pair, $W_{i}=E_{0}/N_{i}$, we obtain

2.2 $$\frac{W_{i}}{E_{g}}=\frac{E_{i}}{E_{g}}+\frac{N_{\mathrm{ex}}}{N_{i}}\times \frac{E_{\mathrm{ex}}}{E_{g}}+\frac{\epsilon }{E_{g}}.$$

The band gap energy is 9.22 eV and 9.28 eV for liquid and solid xenon, respectively, while the ratio $\alpha =N_{\mathrm{ex}}/N_{i}$ is between 0.06 and 0.2. For nuclear recoils, the ratio $N_{\mathrm{ex}}/N_{i}$ is 1, and thus much larger than for electronic recoils. This property is used in dark matter searches via nuclear recoils, discussed in §6. The ratio $W_{i}/E_{g}$ has been calculated as 1.65, in good agreement with measurements, which yield 1.6 [3].

Scintillation arises from excited xenon atoms ${\text{Xe}}^{*}$ (excitons) and from ions ${\text{Xe}}^{+}$, as follows:

(i) $${\mathrm{Xe}}^{*}+\mathrm{Xe}+\mathrm{Xe}\to {\mathrm{Xe}}_{2}^{*}+\mathrm{Xe}$$
  and
  $$\;{\mathrm{Xe}}_{2}^{*}\to 2\mathrm{Xe}+h\nu .$$
(ii) $$\begin{array}{rl} & \;{\mathrm{Xe}}^{+}+\mathrm{Xe}\to {\mathrm{Xe}}_{2}^{+},\\ & \;{\mathrm{Xe}}_{2}^{+}+{\text{e}}^{-}\to {\mathrm{Xe}}^{**}+\mathrm{Xe},\\ & \;{\mathrm{Xe}}^{**}\to {\mathrm{Xe}}^{*}+\mathrm{heat},\\ & \;{\mathrm{Xe}}^{*}+\mathrm{Xe}+\mathrm{Xe}\to {\mathrm{Xe}}_{2}^{*}+\mathrm{Xe},\\ \;\mathrm{and}\; & \;{\mathrm{Xe}}_{2}^{*}\to 2\mathrm{Xe}+h\nu , \end{array}$$

where hν denotes the emitted vacuum-ultraviolet (VUV) photon with wavelength peaked at approximately 175–178 nm. ${\text{Xe}}^{**}\to {\text{Xe}}^{*}+\mathrm{heat}$ corresponds to a non-radiative transition. The excited dimer ${\text{Xe}}_{2}^{*}$ (excimer), at its lowest excited level, is de-excited to the dissociative ground state by the emission of a single VUV photon. This comes from the large energy gap between the lowest excitation and the ground level, forbidding other decay channels such as non-radiative transitions. The scintillation light from pure liquid xenon has two decay components due to de-excitation of spin singlet (${\;}^{1}\Sigma_{u}^{+}$) and spin triplet (${\;}^{3}\Sigma_{u}^{+}$) states of the excited dimer ${\text{Xe}}_{2}^{*}\to 2\text{Xe}+h\nu$ to the ground state (${\;}^{1}\Sigma_{g}^{+}$). The singlet and triplet states refer to the total spin quantum number (s=0 or s=1) of the excited Rydberg electron and the angular momentum owing to the molecular orbit, with the shorter and longer decay shapes being produced by the de-excitation of s=0 states and s=1 states, respectively. The differences of pulse decay shape between different type of particle interactions can be in principle be used to discriminate electronic from nuclear recoils. In practice, this is difficult owing to the small time separation, (2.2±0.3) ns versus (27±1) ns, of the two components.

If we denote $W_{\mathrm{ph}}$ as the mean energy required for the production of a single photon, we can express it as

2.3 $$W_{\mathrm{ph}}=\frac{E_{0}}{N_{\mathrm{ex}}+N_{i}}=\frac{W_{i}}{1+N_{\mathrm{ex}}/N_{i}}=\frac{W_{i}}{1+\alpha },$$

where $N_{\mathrm{ex}},N_{i}$ are the produced number of excitons and electron–ion pairs, respectively, and we assume for simplicity that the efficiencies for an exciton or a recombining electron–ion pair to create a detectable photon are unity such that

2.4 $$N_{\mathrm{ph}}=N_{\mathrm{ex}}+rN_{i},$$

with r being the recombination fraction. If an electric field is applied, one can measure the electrons which do not recombine, with the amount of extracted charge defined as

2.5 $$N_{q}=(1-r)N_{i}.$$

Using above equations, the recombination independent sum can be defined, following [15], as

2.6 $$E_{0}=(N_{q}+N_{\mathrm{ph}})\times W_{\mathrm{ph}}.$$

The recombination independent energy required to produce a single detectable quantum, $N_{q}$ or $N_{\mathrm{ph}}$, is often called the W-value, and $W_{\mathrm{ph}}=W$ is used in the following. This assumes that each recombining electron–ion pair produces an exciton, which leads to a photon. The widely adopted W-value in liquid xenon has been measured as W=(13.7±0.2) eV with a small, 30 g LXe detector using 122 keV γ-rays from an external ${\;}^{57}$Co source [15]. Recently, a lower value of $W=11.5_{-0.3}^{+0.2}$ (syst.) eV was measured with a small TPC using internal sources at energies between 2.8 keV and 42 keV [16], which is consistent with the value measured at the MeV-scale, W=11.5±0.5( syst.) ±0.1( stat.)  eV with a larger detector [17].

The partition between excitation and ionization depends on the density of the electron–ion pairs produced along the track of a particle, and is thus different for nuclear and electronic recoils. The recombination fraction r depends on the applied electric field, as well as on the ionization density in the track. In particular, for nuclear recoils as generated by interactions of fast neutrons or hypothetical weakly interacting massive particles (WIMPs), an energy-dependent quenching is introduced via the Lindhard factor L [18] given as

**Figure 1.**
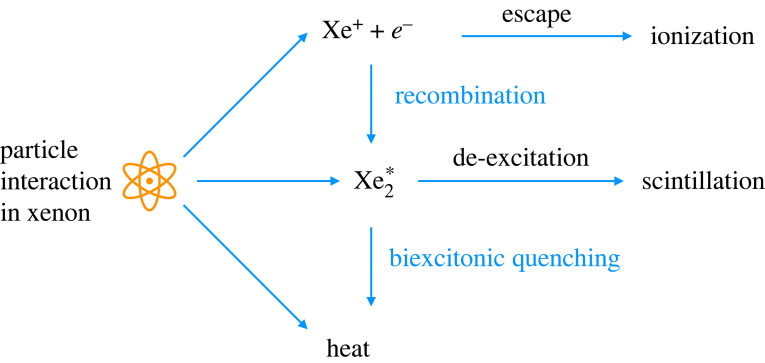
After a particle interacts in a xenon medium, various processes lead to ionization, scintillation and heat. Only scintillation and ionization (via electroluminiscence) are observed in a two-phase Xe-TPC. Biexcitonic quenching [20] is relevant for interaction with high ionization density.

2.7 $$E_{0}=L^{-1}(N_{q}+N_{\mathrm{ph}})W,$$

with L being approximately 0.15--0.2 at nuclear recoil energies in the range 3–100 keV [19]. A schematic view of the signal production after a particle interaction in xenon is shown in figure 1.

## 3. Principles of dual-phase xenon TPCs

Xenon TPCs in astroparticle physics and rare-event searches use either high-pressure gas, or a liquid phase, or liquid and gas phase (also called two-phase or dual-phase) as detection medium; here we focus on the latter. Table 2 lists some properties relevant for building a TPC.

**Table 2.** Properties of liquid xenon as a particle detection medium, relevant for building a TPC.

property (unit)	value
scintillation light yield (at 122 keV) [21]	63 $\mathrm{photons}\;{\mathrm{keV}}^{-1}$
wavelength (peak centred at)	175–178 nm
decay time constants (s=0, s=1)	2.2 ns, 27 ns
refractive index	1.69
electron mobility [22]	$0.29\;{\mathrm{mm}}^{2}\;{\left(\mu s\;V\right)}^{-1}$ ($<100\;V\;{\mathrm{cm}}^{-1}$)
	$0.01\;{\mathrm{mm}}^{2}\;{\left(\mu s\;V\right)}^{-1}$ ($>100\;V\;{\mathrm{cm}}^{-1}$)

**Figure 2.**
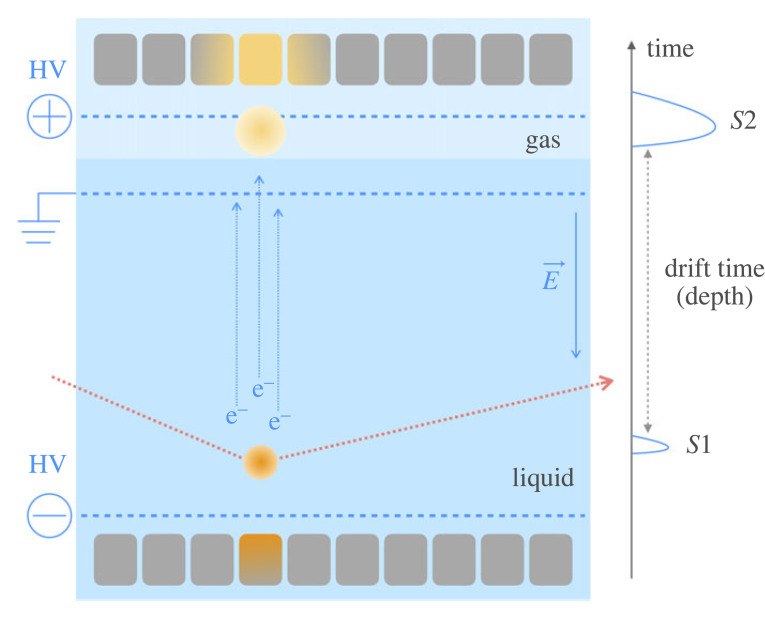
The operation principle of a two-phase xenon TPC. A particle interaction in liquid xenon gives rise to a prompt scintillation signal (S1) and a delayed, amplified proportional scintillation signal (S2). The latter is caused by ionization electrons, which are drifted in a homogeneous electric field (of a few $100\;V\;{\mathrm{cm}}^{-1}$) and extracted into the gas phase above the liquid with a higher electric field, typically $10\;\mathrm{kV}\;{\mathrm{cm}}^{-1}$. The drift field is produced between the cathode at negative potential and a grounded gate grid in the liquid, while the extraction field is obtained by means of the anode placed above the gate in the gas phase. Both S1 and S2 signals are observed with photosensor arrays placed on the bottom and top of the TPC.

The operation principle of a two-phase TPC is easy to grasp, and it is shown schematically in figure 2. An interaction within the active volume of a detector will create ionization electrons and prompt scintillation photons. The prompt scintillation signal (S1) is detected with two arrays of photosensors, one in the liquid phase on the bottom and one in the gas phase at the top. The electrons drift in the pure liquid under the influence of an external electric field, are then accelerated by a stronger field and extracted into the vapour phase above the liquid, where they generate proportional scintillation, or electroluminiscence. The delayed, proportional scintillation signal (S2) is observed by the same photosensor arrays. The array immersed in the liquid collects the majority of the prompt signal, which is totally reflected at the liquid–gas interface. The ratio of the two signals is different for nuclear recoils, such as from fast neutron interactions or hypothetical WIMPs and electronic recoils produced by β- and γ-rays. This provides the basis for background discrimination in dark matter detectors. Since electron diffusion in the ultra-pure liquid is small (albeit non-negligible, see §5e), the proportional scintillation photons carry the x–y information of the interaction site. With the z-information from the drift time measurement, the TPC yields a three-dimensional event localization, enabling fiducial volume selections and differentiation between single- and multiple-scatters in the active volume.

## 4. Energy calibration and resolution of xenon TPCs

An advantage of two-phase Xe-TPCs is their relatively good energy resolution, achieved by taking a linear combination of the two anti-correlated signals, S1 and S2. To calibrate the energy scale, mono-energetic lines from external (e.g. ${\;}^{57}\mathrm{Co}$, ${\;}^{137}\mathrm{Cs}$, ${\;}^{228}\mathrm{Th}$, etc.) and internal (${\;}^{83m}\mathrm{Kr}$, ${\;}^{37}\mathrm{Ar}$) calibration sources are used. Additional signals are provided by neutron-activated xenon (${\;}^{129m}\mathrm{Xe}$, ${\;}^{131m}\mathrm{Xe}$) and the radioactivity of detector components (e.g. ${\;}^{60}\mathrm{Co}$, ${\;}^{208}\mathrm{Tl}$). The mean detected S1 and S2 signals per produced photon and ionization electron are denoted as $g_{1}$ and $g_{2}$, respectively: $g_{1}=S1/N_{\mathrm{ph}}$, $g_{2}=S2/N_{q}$, also called the total photon detection efficiency and the charge amplification factor. We can then express equation (2.6) as

4.1 $$E_{0}=(N_{\mathrm{ph}}+N_{q})\times W=\left(\frac{S1}{g_{1}}+\frac{S2}{g_{2}}\right)\times W.$$

Rearranging this equation yields

4.2 $$\frac{S2}{E_{0}}=\frac{g_{2}}{W}-\frac{g_{2}}{g_{1}}\frac{S1}{E_{0}}.$$

**Figure 3.**
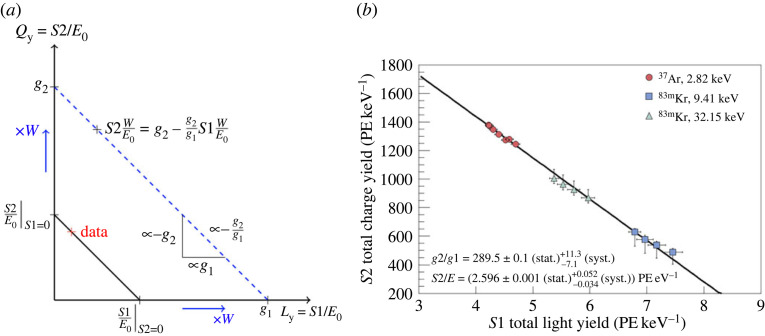
(*a*) Schematic of a Doke plot in charge ($Q_{y}$) versus light ($L_{y}$) yield for an interaction energy $E_{0}$. Measurements of the charge and light yield at different electric drift fields or interaction energies yield data along the solid line. The dashed line, showing the relation to the gain parameters $g_{1}$ and $g_{2}$, is obtained by scaling with the W-value. (*b*) Normalized scintillation and ionization signals at low energies for different drift fields in the range $80\text{--}968\;V\;{\mathrm{cm}}^{-1}$ for a small TPC, Xurich. Figures from [16].

Since W and $E_{0}$ (for a given mono-energetic signal) are known, we can determine $g_{1}$ and $g_{2}$ from the measured S1 and S2 signals at several energies, in a so-called ‘Doke plot’, a schematic of which is shown in figure 3*a*.

If we plot $S2/E_{0}$ (charge yield, $Q_{y}$) as a function of $S1/E_{0}$ (light yield, $L_{y}$) , we can obtain $g_{2}$/$g_{1}$ from the slope, and $g_{2}$ from the intercept of the fitted line.

Another method to measure $g_{1}$ and $g_{2}$ by varying the signal ratio between S1 and S2 is to use different electric fields with one or several calibration sources, exemplified in figure 3*b*. The quantities $g_{1}$ and $g_{2}$ are thus detector dependant, for they include the detector’s efficiency to detect the light and charge signals. Finally, the S2 gain can be determined directly from the mean size of the S2 signal for single electrons extracted into the gas phase. Relative energy resolutions (σ/μ) at the level of 4–6% at energies of a few tens of keV and 2–3% at energies of a few 100 keV were reached in xenon TPCs [10,23,24]. At higher energies, relevant for the neutrinoless double beta decay of ${\;}^{136}\mathrm{Xe}$, relative resolutions of 0.67%, 0.8% and 1.2% around 2.5 MeV were obtained by LZ [25], XENON1T [26] and EXO-200 [27], respectively.^1^

A comprehensive framework to simulate scintillation and ionization yields and resolutions as a function of interaction type, energy and electric field in a TPC is the Noble Element Simulation Technique (NEST) [31]. The code, based on phenomenological models informed by a vast array of data, also allows for simulating detector specific effects, once the primary and secondary scintillation gains, as well as the drift field, are specified. NEST models are regularly updated when new data becomes available.

## 5. Past, current and future experiments

The first dual-phase Xe-TPCs that set competitive constraints on WIMP scatters off nuclei were those of the ZEPLIN and XENON programmes, in particular ZEPLIN-II and ZEPLIN-III at Boulby Mine in the UK (2007–2008), and XENON10 at LNGS in Italy (2006). These initial detectors evolved into LUZ and LUX-ZEPLIN at SURF, USA, and XENON100, XENON1T and XENONnT at LNGS. In parallel, PandaX-I and PandaX-II were constructed at the China Jinping Underground Laboratory (CJPL), with first results in 2014, followed by PandaX-4T.

**Figure 4.**
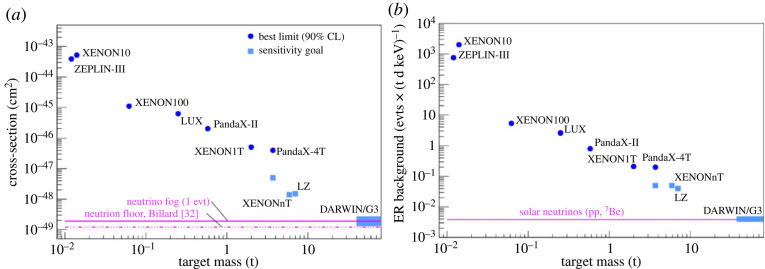
Evolution of the sensitivity to SI WIMP-nucleon interactions (*a*) and electronic recoil background level (*b*) of dual-phase Xe-TPCs as a function of target mass. We note the logarithmic scales. Figures from [32].

Starting with total masses at the few kilogram and later 100 kg scale, the detectors evolved and reached target masses at the tonne- and more recently multi-tonne scale. Concomitantly, the background levels in the most inner regions constantly decreased, with now unprecedented electronic recoil levels around $15\;\mathrm{events}\;{\left(t\;y\;\mathrm{keV}\right)}^{-1}$ in the energy region below 100 keV. Figure 4 shows the remarkable evolution of the sensitivity to WIMPs (*a*) and of the background level (*b*) as a function of target mass. The figures cover a span of approximately 20 years.

The current generation of detectors use several tons of LXe: LZ [33], PandaX-4T [34] and XENONnT [35] have total (target) LXe masses of 10 t (7 t), 5.6 (3.7 t) and 8.6 t (5.9 t), respectively. While their overall TPC design is rather similar, with cylindrical, PTFE enclosed target regions viewed by two arrays of 3-inch diameter Hamamatsu R11410 PMTs, their detailed realization differs in many technical aspects, some of which are described in the following.

### (a) The LUX-ZEPLIN experiment

The LUX-ZEPLIN (LZ) experiment is located at the Sanford Underground Research Facility (SURF) in South Dakota, USA. The inner, xenon detector, is composed of a TPC and a veto, called Xe Skin, enclosed in a double-walled, low-background titanium cryostat. The dark matter target in the TPC contains 7 tonnes of LXe in a cylindrical volume which measures 1.5 m in diameter and height. The vapour phase above the liquid is 8 mm thick, and the site of the S2 signal production. The TPC is lined with PTFE and instrumented with 494 3-inch Hamamatsu R11410-22 PMTs arranged in two arrays. The Xe Skin detector, around and underneath the TPC with 2 tonnes of instrumented liquid with 93 1-inch and 38 2-inch PMTs, acts as an anti-coincidence scintillation detector, especially effective for gamma radiation. The cryostat is surrounded by an outer detector, a system of acrylic tanks containing 17 t of Gd-loaded liquid scintillator, placed in a 238 t tank of ultra-pure water. The tank is equipped with 120 8-inch PMTs which record both outer detector and the water Cherenkov signals. The LZ experiment has presented first results on WIMP dark matter for an exposure of 60 live days with 5.5 t of liquid xenon fiducial mass. The main electronic recoil background comes from ${\;}^{214}\mathrm{Pb}$ β-decays, with a ${\;}^{222}\mathrm{Rn}$ concentration in LXe of $3.3\;\mu \mathrm{Bq}\;{\mathrm{kg}}^{-1}$ and from ${\;}^{37}\mathrm{Ar}$ decays, with a half-life of 35 days. The xenon gas was purified of krypton using gas chromatography, with a ${\;}^{\text{nat}}\mathrm{Kr}$ rate of (0.14±0.02) ppt. The data were consistent with a background-only hypothesis and new upper limits on spin-independent and spin-dependent WIMP-nucleon cross-sections for WIMP masses above 9 GeV were set, with the most stringent constraint at $6.5\times 10^{-48}\;{\mathrm{cm}}^{2}$ for a mass of $30\;\text{GeV}\;{\text{c}}^{-2}$ [36]. The LZ experiment continues to take science data at SURF, with a projected sensitivity for spin-independent (SI) WIMP-nucleon cross-sections of $1.5\times 10^{-48}\;{\mathrm{cm}}^{2}$ for a $40\;\text{GeV}\;{\text{c}}^{-2}$ WIMP (at 90% C.L.) and an exposure of 20 t y.

### (b) The PandaX-4T experiment

The PandaX-4T experiment is located at CJPL, built deep underground in the Jinping Mountains of Sichuan. The inner detector is a TPC with 3.7 tonnes of liquid xenon, contained by 24 PTFE wall panels and observed by 169 and 199 R11410-23 three-inch PMTs in the top and bottom, respectively. The TPC and xenon are contained in a double-walled, low-background stainless steel cryostat, placed at the centre of a 10 m diameter and 13 m tall tank filled with ultra-pure water. The space between the TPC field cage and the cryostat is instrumented with two rings of one-inch R8520 Hamamatsu PMTs, for an active xenon veto. The xenon inventory was distilled for krypton through a distillation tower prior to and during the commissioning run, which included 95 days of stable data taking, for a ${\;}^{\text{nat}}\mathrm{Kr}$ rate of (0.33±0.02) ppt. The dominant electronic recoil background was due to β-decays from ${\;}^{3}H$, with an average concentration of $5\times 10^{-24}\;\mathrm{mol}\;{\mathrm{mol}}^{-1}$ in Xe, followed by β-decays from ${\;}^{214}\mathrm{Pb}$, with a ${\;}^{222}\mathrm{Rn}$ concentration between 4.2 and $5.9\;\mu \mathrm{Bq}\;{\mathrm{kg}}^{-1}$. The commissioning data were used for a WIMP search, with an exposure of 0.63 tonne year. No dark matter candidate events were observed above the expected background, with the most stringent upper limit on SI WIMP-nucleon cross-sections of $3.8\times 10^{-47}\;{\mathrm{cm}}^{2}$ for a $40\;\text{GeV}\;{\text{c}}^{-2}$ WIMP [34]. Because of the tritium contamination of the xenon, probably originating from a ${\text{CH}}_{3}T$ calibration of the previous detector, the collaboration undertook a tritium removal campaign, before commencing the taking of physics data. The goal is to reach another order in magnitude improvement in sensitivity with an exposure of 6 t y.

### (c) The XENONnT experiment

The XENONnT experiment is located at the INFN Laboratori Nazionali del Gran Sasso (LNGS) in Italy. The TPC contains 5.9 t of LXe, enclosed by a PTFE cylinder with a diameter of 1.33 m and a height of 1.49 m. A total of 494 Hamamatsu R11410-21 3-inch PMTs distributed in a top and a bottom array view the sensitive volume. A double-walled, low-radioactivity stainless steel cryostat houses the TPC and is filled with 8.5 t of LXe. The cryostat is placed in a 700 t water Cherenkov muon veto, 10.2 m high and 9.6 m in diameter, equipped with 84 8-inch PMTs. A volume of $33\;m^{3}$ of water surrounding the cryostat and delimited by octagonal caps and side reflectors made of high-reflectivity expanded PTFE is used as a neutron veto, seen by 120 8-inch PMTs. In the first physics run, the vetos contained ultra-pure water, while later the water was doped with Gd to increase the neutron veto efficiency. XENONnT had accumulated 95.1 days of data during its first science run. A high-throughput radon distillation column was continuously operated to remove radon from the gaseous xenon in the TPC, while a krypton distillation column facilitated a ${\;}^{\text{nat}}\mathrm{Kr}$ concentration of less than 50 ppq. In XENONnT, a novel LXe purification system was installed, with a capability to reach a very high removal rate of electronegative impurities in a short time, with an electron drift lifetime above 10 ms during the science run. The dominant electronic recoil background in the first science run was due to ${\;}^{214}\mathrm{Pb}$ β-decays, with a ${\;}^{222}\mathrm{Rn}$ concentration of $1.8\;\mu \mathrm{Bq}\;{\mathrm{kg}}^{-1}$. A blind analysis revealed no significant excess from WIMPs in an exposure of 1.09 t y, with the lowest upper limit on SI WIMP-nucleon cross-sections of $2.58\times 10^{-47}{\mathrm{cm}}^{2}$ at $28\;\text{GeV}\;{\text{c}}^{-2}$ (90% C.L.) [37]. The ER background of $(15.8\pm 1.3)\;\mathrm{events}\;{\left(t\;y\;\mathrm{keV}\right)}^{-1}$ was the lowest in any dark matter detector, and in particular five times lower than in XENON1T. XENONnT continues to take data at LNGS, with a further reduced ${\;}^{222}\mathrm{Rn}$ concentration of $0.8\;\mu \mathrm{Bq}\;{\mathrm{kg}}^{-1}$, using the radon distillation system with combined gaseous and liquid xenon flow.

### (d) Next-generation projects

DARWIN, first proposed around 2011 [38], is a next-generation observatory with 40 t of LXe in the TPC (50 t total). In the baseline design, the cylindrical TPC, with 2.6 m diameter and 2.6 m height, is placed in a low-background, double-walled titanium cryostat, surrounded by active neutron and muon vetos. Two photosensor arrays with a total of 1910 3-inch PMTs are located at the top and bottom of the TPC, which is lined with high-reflectivity PTFE, surrounded by 92 copper field shaping rings [39]. The research and development for new type of photosensors, which could potentially replace the 3-inch PMTs, is ongoing. The sensors include VUV-sensitive silicon photomultipliers, 2-inch×2-inch flat panel PMTs (R12699), and hybrid photosensors. Recently, the LZ, XENON and DARWIN collaboration joined forces to form the XLZD consortium [40], with the goal of jointly constructing and operating the next-generation observatory. The size and scope of the detector might be enlarged, compared to DARWIN, with a 3 m×3 m TPC containing 60 t of LXe (75 t in total). The larger mass would allow for a 3-σ WIMP discovery at a SI cross-section of 3$\times 10^{-49}{\mathrm{cm}}^{2}$ at $40\;\text{GeV}\;{\text{c}}^{-2}$ mass (90% C.L.), and would also increase the sensitivity to the neutrinoless double-beta decay of ${\;}^{136}\mathrm{Xe}$. The science potential of a large, dual-phase xenon detector is detailed in [32].

PandaX-xT is the next step in the PandaX programme at CJPL, with greater than 30 t of LXe target in the TPC. Two arrays of Hamamatsu R12699 2-inch PMTs will view the Xe volume. Compared to the 3-inch tubes used in current TPCs, these new sensors have the advantage of lower radioactivity, faster time response and the possibility of multi-anode readout, with four independent channels per unit. The inner cryostat vessel will be made of ultra-pure copper, and the space between the inner and outer vessel will contain an active veto. PandaX-xT aims for a 200 t y exposure for WIMPs, and, similar to DARWIN/XLZD, for a broad science reach [41].

### (e) Technological challenges

**Figure 5.**
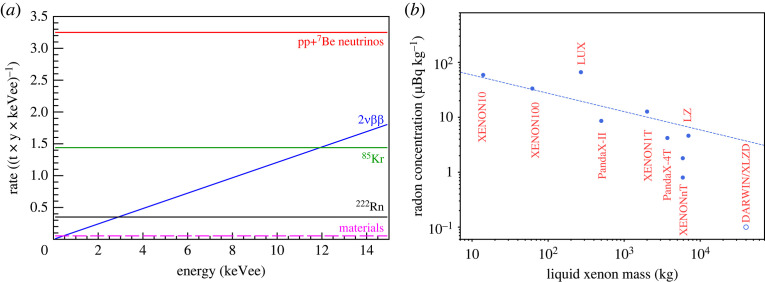
(*a*) Differential energy spectra of electronic recoil background sources in DARWIN. The dominating contribution is from pp- and ${\;}^{7}\mathrm{Be}$ solar neutrinos. Figure from [42]. (*b*) The evolution of ${\;}^{222}\mathrm{Rn}$ concentration in two-phase Xe-TPCs (measured values, blue dots), together with the expected decrease from the surface-to-volume ratio (dashed line, $x^{-1/3}$). The goal for next-generation TPCs (open circle) is also shown.

As we have seen, dual-phase Xe-TPCs were successful in gradually scaling up their target mass from a few kg to multi-tons, and concomitantly reducing the background level for each iteration, while maintaining a low-energy threshold of approximately 1 keV for electronic recoils. Notwithstanding, the construction of next-generation detectors with multi-ten-tons target masses poses multiple technological challenges. The background goals are such that electronic and nuclear recoil rates are below the ones from irreducible astrophysical neutrino interactions. This requirement then also sets the goals for the intrinsic ${\;}^{222}\mathrm{Rn}$ and ${\;}^{85}\mathrm{Kr}$ concentrations: the background from the decay of these isotopes should be significantly lower than the solar pp-neutrino elastic scattering rate, as shown in figure 5*a*. This translates into $0.1\;\mu \mathrm{Bq}\;{\mathrm{kg}}^{-1}$ for ${\;}^{222}\mathrm{Rn}$^2^ and 0.1 ppt for ${\;}^{\text{nat}}\mathrm{Kr}$, assuming a ${\;}^{85}\mathrm{Kr}{/}^{\text{nat}}\mathrm{Kr}$ ratio of $2\times 10^{-11}\;\mathrm{mol}\;{\mathrm{mol}}^{-1}$. ${\;}^{\text{nat}}\mathrm{Kr}$ concentrations of less than 50 ppq were already achieved by cryogenic distillation [43], while for ${\;}^{222}\mathrm{Rn}$ a reduction factor of approximately 10 compared to the current value of $0.8\;\mu \mathrm{Bq}\;{\mathrm{kg}}^{-1}$ in XENONnT is still needed, see figure 5*b*. This calls for a high liquid xenon throughput (close to $1\;t\;h^{-1}$) with efficient cooling power based on cryogenic heat pumps and radon-free heat exchangers. Kryogenic distillation alone is not sufficient: it must go hand in hand with selection of low radon emanation materials and new coating techniques to prevent radon emanation from surfaces.

Other challenges related to the liquid target are the continuous purification for electronegative impurities and water, to maintain high charge and light yields, as well as new solutions for reliable xenon storage and recuperation at large scales. Liquid phase purification powered by a liquid xenon pump, as demonstrated in [44], was used to achieve an electron drift lifetime above 10 ms in approximately 8.6 t of xenon in XENONnT. A system capable of handling 30 t of xenon in liquid phase, including long-term storage and transfer of the cryogenic liquid between storage module and detectors was constructed for PandaX-xT [41].

Regarding the detector design: electrodes with large (greater than 2.5 m) diameters, with high transparency, minimal sagging and low spurious electron emission, as well as high-voltage feed-throughs that can safely deliver 50 kV or more to the cathode must be developed. The LZ collaboration successfully built custom-woven wire-mesh grids with 1.5 m diameter, produced with an in-house built loom to weave the wire meshes [45]. Finally, the cryostat design must be such as to ensure stability, while reducing as much as possible the amount of material, and thus gamma and neutron emitters in proximity to the TPC.

To address a series of challenges related to constructing and operating next-generation Xe-TPCs, several large-scale demonstrators have been built, in particular Xenoscope, which includes a 2.6 m tall TPC [22,46] and Pancake, to deploy a 2.6 diameter TPC [47].

## 6. Sensitivity to dark matter candidates

**Figure 6.**
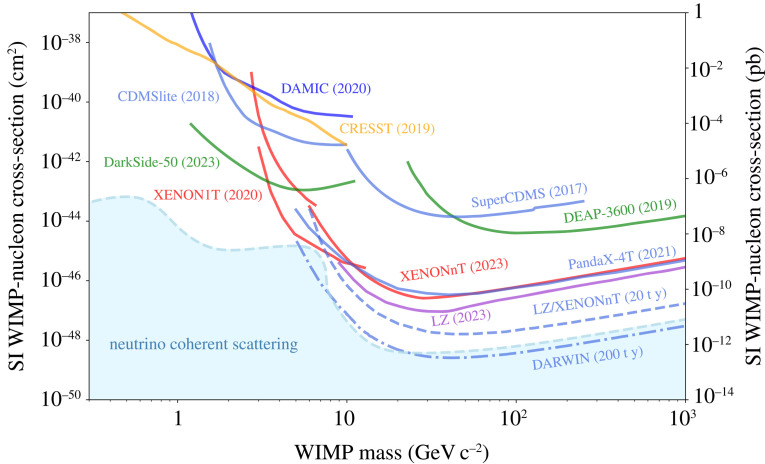
Exclusion limits (solid) on the spin-independent WIMP-nucleon cross-section from dual-phase Xe-TPCs as well as from other technologies. Projections for LZ and XENONnT (dashed) and for DARWIN/XLZD (dashed-dotted) are also shown. The region where a distinction between a dark matter signal and astrophysical neutrinos will be challenging, albeit not impossible [48], is shown in blue. Figure updated from [49].

The current generation of detectors did not find evidence for WIMP dark matter, but were able to set the world’s most stringent limits on WIMP-nucleon interactions over a wide range of particle masses and thus to exclude a large range of possibilities. These results were from very first data and the experiments are yet to reach their design exposures of 20 t y, see figure 6. The search for WIMP dark matter is thus ongoing, with the goal of reaching a sensitivity for the SI WIMP-nucleon cross-section of approximately $1.5\times 10^{-48}\;{\mathrm{cm}}^{2}$ at $40\text{--}50\;\text{GeV}\;{\text{c}}^{-2}$ mass [33,35]. While dark matter particles are thought to be electrically neutral, they could posses tiny electromagnetic couplings to photons, allowed by all the data. The PandaX collaboration looked for nuclear recoils generated by potential dark matter–photon interactions, setting tight constraints on the magnitudes of millicharge, magnetic dipole, electric dipole and anapole moments, as well as first constraints on the charge radius of dark matter [50].

**Figure 7.**
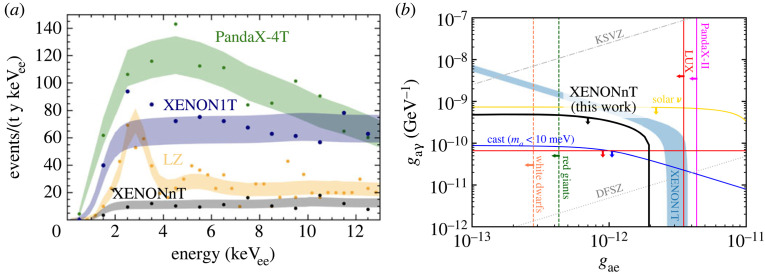
(*a*) Observed event rates at low energies in XENONnT (grey), compared with XENON1T (blue), as well as with LZ (orange) and PandaX-4T (green). The shaded areas indicate the estimated backgrounds. Figure by J. Ye, Columbia University. (*b*) Constraints on solar axion couplings to photons and electrons from XENONnT (black), the XENON1T result (blue), as well as other direct and indirect constraints [52].

Because of unprecedented low electronic recoil backgrounds and low-energy thresholds, high-sensitivity electronic recoil searches have become feasible. These include searches for dark matter electron scattering with particle candidates from a hidden sector, searches for keV-scale axion-like-particles (ALPs) and dark photons via absorption in LXe, and searches for solar axions. In 2020, XENON1T reported a surprising excess of events in the energy region 1–7 keV, which could have been due to solar axions (with a statistical significance of 3.4σ), galactic ALPs, or a neutrino magnetic moment. The excess, however, could also have been caused by minute amounts of tritium in the xenon, at the level of $6\times 10^{-25}\;\mathrm{mol}\;{\mathrm{mol}}^{-1}$, or less than three tritium atoms per kilogram of LXe [51]. With the accumulated number of events, it was not possible to confirm or reject the tritium hypothesis with XENON1T, and a larger detector with lower backgrounds was urgently needed. With data from its first science run, and an electronic recoil rate five times below the one of its predecessor, XENONnT did not see any excess above background. The rate and shape agreed well with predictions and, between 1 and 10 keV, were dominated by ${\;}^{214}\mathrm{Pb}$ β-decays from ${\;}^{222}\mathrm{Rn}$ dissolved in the LXe, followed by interactions of pp solar neutrinos [52].^3^ A comparison between low-energy event rates in XENON1T, showing the excess, XENONnT, PandaX-4T and LZ is shown in figure 7*a*. Among other results, XENONnT set new constraints on the coupling of solar axions to electrons and photons, via the axio-electric and inverse Primakoff effects, respectively, shown in figure 7*b* [52]. Sensitivity projections for LZ to new physics via low-energy electronic recoils, including mirror and leptophilic dark matter, are detailed in [53].

**Figure 8.**
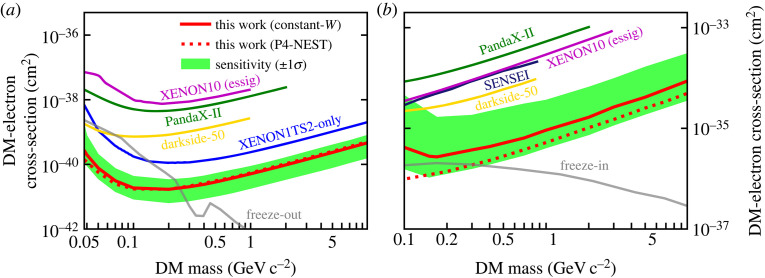
Upper limits on the cross-section for dark matter electron scatters, assuming a heavy ((*a*), with a dark matter form factor = 1) and light ((*b*), with a dark matter form factor $∼1/q^{2}$) mediator, from the PandaX-4T experiment (red), as well as other constraints. Dual-phase Xe-TPCs thus start testing theoretical predictions, shown as grey bands for different dark matter production scenarios. Figures from [54].

Looking at ionization-only signals allows for a further reduction of the energy threshold, given the much higher efficiency to detect an ionization electron compared to a primary scintillation photon (typically 90% versus 10%). While this gain comes at the expense of higher backgrounds, it allows nonetheless to set the most stringent limits on light dark matter electron interactions at masses from a few tens of MeV to a few GeV. This is illustrated in figure 8, which shows recent result from PandaX-4T [54] as well as from previous searches. Dual-phase Xe-TPCs thus start probing theoretical predictions for different dark matter production scenarios in hidden sectors.

## 7. Second-order weak interactions

Large TPCs using liquid xenon in its natural isotopic abundance are sensitive to double weak decays, such as the double-beta decay of ${\;}^{134}\mathrm{Xe}$ and ${\;}^{136}\mathrm{Xe}$, with Q-values of 825.8 keV and 2457.8 keV, respectively, and the double electron capture process in ${\;}^{124}\mathrm{Xe}$ and ${\;}^{126}\mathrm{Xe}$, at Q-values of 2857 keV and 920 keV. In particular, the observation of the neutrinoless decay modes

7.1 $$(Z,A)\to (Z+2,A)+2\;e^{-}$$

and

7.2 $$(Z,A)+2\;e^{-}\to (Z-2,A),$$

would be evidence for lepton number violation involving Majorana neutrinos. The observed standard model processes with two neutrinos (so far, 2νββ in ${\;}^{136}\mathrm{Xe}$ and 2νECEC in ${\;}^{124}\mathrm{Xe}$) allow for comparison to theoretical half-life predictions from nuclear structure calculations in different nuclear physics models, as well as for new physics searches (see, e.g. [55] for a recent overview).

In its electronic recoil channel, the XENON1T experiment observed for the first time the 2νECEC process in ${\;}^{124}\mathrm{Xe}$ by detecting the simultaneously emitted K-shell X-rays/Auger electrons of the daughter atom ${\;}^{124}\mathrm{Te}$ with a combined energy of 64.33 keV, which is twice the K-shell binding energy. With a half-life of $(1.1\pm 0.2_{\text{stat}}\pm 0.1_{\text{sys}})\times 10^{22}$ y, this is the slowest process ever measured directly [10,11]. LZ, PandaX-4T and XENONnT will improve upon these results and will also search for the $2\nu \beta^{+}$EC decay. This channel has a distinct signature owing to the two 511 keV gammas emitted after the positron annihilates with an electron in the medium. With a predicted half-life of approximately $1.6\times 10^{23}$ y [56] its first observation is within reach of these running experiments, which will also measure the half-life and in particular also the shape of the 2νββ-decay of ${\;}^{136}\mathrm{Xe}$ with high statistics and at low energies not previously accessed.

**Figure 9.**
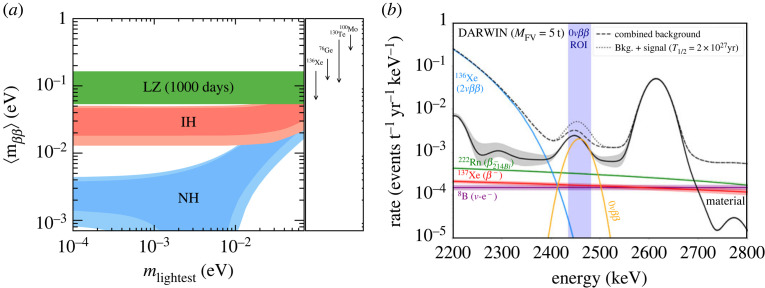
(*a*) Projected sensitivity of LZ (green band) to the effective Majorana neutrino mass, as a function of the lightest neutrino mass eigenstate. The red and blue contours show the allowed parameter space at 1-sigma for the inverted and normal neutrino mass ordering scenarios, respectively. Figure from [58]. (*b*) Predicted background spectrum in DARWIN around the region-of-interest for 0νββ-decay for 5 t LXe fiducial mass. A hypothetical signal of $0.5\;\mathrm{events}\;y^{-1}$, corresponding to a half-life of $2\times 10^{27}$ y, is shown for comparison. Figure updated from [60].

Finally, the current generation of detectors will set constraints on the 0νββ-decays of ${\;}^{136}\mathrm{Xe}$ and ${\;}^{134}\mathrm{Xe}$, as predicted or shown in [11,57–59], see figure 9*a*. In spite of relative energy resolutions (σ/μ) of 0.8% and 0.67% [25,26] at high energies, the attainable half-lives are not competitive to dedicated experiments such as KamLAND-Zen [61] and the proposed nEXO [62]. Notwithstanding, the analyses will deliver proof-of-principle methods towards higher sensitivity searches in the DARWIN/XLZD and PandaX-xT detectors. As an example DARWIN, with 40 t of natural xenon in the TPC, is predicted to achieve a sensitivity of $3\times 10^{27}$ y (90% C.L.) after 10 years of operation [60]. The predicted background spectrum around the Q-value of the decay, together with a hypothetical signal, is shown in see figure 9*b*. With an enlarged xenon mass of 60 t in the TPC, as advocated by XLZD, a half-life sensitivity of $∼5\times 10^{27}$ y (90% C.L.) could be reached after 10 years, allowing full investigation of the inverted mass ordering scenario for neutrinos.

## 8. Neutrino detection

Recently, dual-phase Xe-TPCs have reached target scales which allows for the exploration of a variety of astrophysical neutrino sources. The main detection channels are elastic neutrino–electron scattering and coherent elastic neutrino-nucleus scattering:

8.1 $$\nu_{x}+e^{-}\to \nu_{x}+e^{-},$$

and

8.2 $$\nu_{x}+(Z,A)\to \nu_{x}+(Z,A),$$

where $\nu_{x}$ stands for all three neutrino flavours, $\nu_{e},\nu_{\mu },\nu_{\tau }$. The latter process, in which the neutrino scatters off a nucleus via Z-boson exchange and the nucleus recoils as a whole, is coherent up to neutrino energies of approximately 50 MeV.

The observation of elastic neutrino–electron scattering of solar pp and ${\;}^{7}\mathrm{Be}$ neutrinos will allow the measurement of pp and ${\;}^{7}\mathrm{Be}$ fluxes with 0.15% and 1% statistical precision for a 300 t y exposure, with rates of $365\;\mathrm{events}\;{\left(t\;y\right)}^{-1}$ and $140\;\mathrm{events}\;{\left(t\;y\right)}^{-1}$, respectively. This will imply a first measurement of the electron neutrino survival probability with 4% relative uncertainty, as well as of the weak mixing angle, with 5% relative uncertainty, at energies below 200 keV [63]. The main backgrounds for these measurements are ${\;}^{222}\mathrm{Rn}$ decays, with a required concentration of $0.1\;\mu \mathrm{Bq}\;{\mathrm{kg}}^{-1}$, and the 2νββ decay of ${\;}^{136}\mathrm{Xe}$. A first detection of solar pp neutrinos will already be achievable with the current generation of detectors. As we have seen, the solar pp rate was almost half the rate from ${\;}^{214}\mathrm{Bi}$ β-decays in XENONnT’s first science run, in the energy range 1–10 keV. It is also interesting to note that, for the first time in a dark matter detector, the neutrino-induced rate is a factor of approximately 1.6 above the one from the radioactivity of detector materials [52].

Coherent elastic neutrino-nucleus scattering (CEνNS) was proposed 50 years ago by Freedman [64], who wrote: ‘The experiments are very difficult, although the estimated cross-sections (approximately $10^{-38}\;{\mathrm{cm}}^{2}$ on carbon) are favourable.’^4^ The experiments were difficult indeed, and the process was only observed four decades later by the COHERENT collaboration with a CsI(Na) scintillating crystal [66], and later also with a liquid argon detector [67]. COHERENT uses neutrinos from pion decays at rest, produced at the Spallation Neutron Source at Oak Ridge National Laboratory. Thus, to this day, CEνNS has never been observed on xenon and never with ‘naturally occurring’ neutrinos.

**Figure 10.**
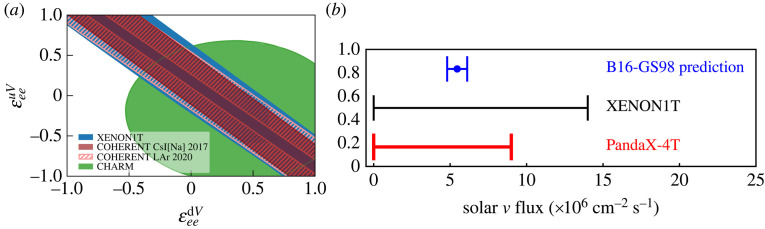
(*a*) Constraints on non-standard vector couplings between electron neutrinos and up/down quarks from XENON1T (blue), COHERENT (light and dark red) and CHARM (green). Shown is the ratio with respect to the SM expectation: the presence of NSI would result in an enhancement or a suppression of the CEνNS rate. Figure from [69]. (*b*) Constraints on the ${\;}^{8}B$ solar neutrino flux via CEνNS from XENON1T (black), PandaX-4T (red) as well as the standard solar model (B16-GS98) prediction (blue). Figure from [70].

The cross-section is proportional to the number of neutrons squared, hence heavy nuclei are ideal to observe astrophysical neutrinos via this reaction. For ${\;}^{8}B$ solar neutrinos, approximately 99% of events are at nuclear recoil energies below 3 keV in a xenon detector, which poses one of the main difficulties in observing CEνNS-induced events. Firstly, the detection efficiencies in Xe-TPCs are relatively low at these energies (in particular if both S1 and S2 signals are required) and secondly, the light and charge yields have large associated uncertainties.^5^ In addition, the combinatorial background (random pairing of S1 and S2 events) is largest at low energies [69], and the problem is more severe for larger detectors with increased electron drift time (and possibly higher rates of spurious events). Nonetheless, the current generation of multi-ton xenon TPCs aim to observe ${\;}^{8}B$ solar neutrinos for the first time. Figure 10 shows constraints on the ${\;}^{8}B$ neutrino flux from XENON1T [69] and PandaX-4T [70], as well as constraints on non-standard neutrino interactions from XENON1T and COHERENT. Next-generation experiments will carry out precision measurements of ${\;}^{8}B$ neutrinos induced CEνNS, and, with an event rate of approximately 90 events per tonne and year [71] they will probe non-standard neutrino interactions and provide an independent measurement of the solar ${\;}^{8}B$ neutrino flux [32].

**Figure 11.**
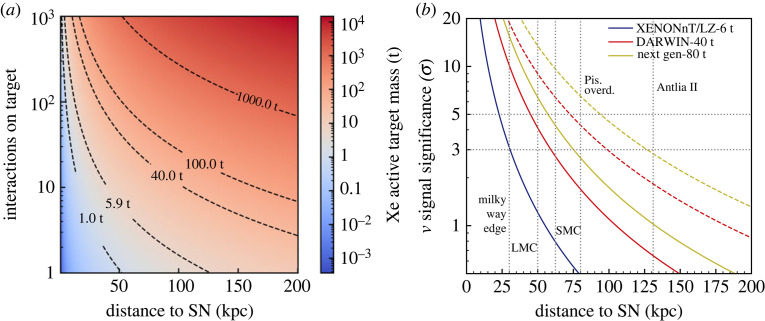
Number of SN ν interactions in a Xe detector for a range of target masses (*a*) and detection significance (*b*) as a function of distance to the SN, for a $27M_{⊙}$ progenitor. The solid (dashed) curves in the right plot, also showing the sensitivity of XENONnT/LZ, assume current (decreased by a factor of 10) level of backgrounds. Figures from [73].

Apart from solar neutrinos, two-phase Xe-TPCs will be able to detect CEνNS-induced events from supernova (SN) neutrinos. During the collapse of a star, approximately 99% of the gravitational binding energies of the proto-neutron star goes into neutrinos of all flavours, with energies up to tens of MeV. Given the nature of the interaction process, xenon detectors are sensitive to all neutrino flavours with few events per tonne expected for a core-collapse SN at a distance of 10 kpc. Assuming a SN with $27M_{⊙}$ progenitor at a distance of 10 kpc, 700 events are expected in the DARWIN detector with 40 t of LXe [72]. This number increases for a larger mass, as does the significance of a SN detection, for a given distance, as illustrated in figure 11.

Current xenon experiments participate in the supernova early warning system (SNEWS) network [74], which prepares and provides an early warning system for galactic SN to facilitate the observation of optical counterparts. Finally, next-generation Xe-TPCs will start constraining the diffuse supernova background (DSNB). Understanding core-collapse SN depends on probing the DSNB with all neutrino flavours, and currently only upper limits on the $\nu_{e},{\bar{\nu }}_{e}$ flux the SNO and Super-Kamiokande experiments exist, approximately $19\;{\mathrm{cm}}^{-2}\;s^{-1}$ and $2.7\;{\mathrm{cm}}^{-2}\;s^{-1}$, respectively. Limits on the fluxes of $\nu_{\mu ,\tau },{\bar{\nu }}_{\mu ,\tau }$ are much weaker, approximately $10^{3}\;{\mathrm{cm}}^{-2}\;s^{-1}$, and a large xenon detector could improve these by about a factor of 100. While this would help in constraining SN models, the actual detection of the DSNB remains challenging, even with a 1000 t y exposure [75].

## 9. Conclusion

Dual-phase Xe-TPCs are relatively new detectors in the landscape of astroparticle physics experiments. Developed in the early twenty-first century primarily to search for dark matter in the form of WIMPs, they soon surpassed other technologies in terms of their sensitivity to WIMP-nucleon interactions over a large range of WIMP masses [76]. Almost 20 years later, detectors using several tonnes of liquid xenon can detect signals down to a few quanta (photons and electrons) with unprecedented low background rates. These detectors will soon observe for the first time solar neutrinos at energies not previously explored, both via neutrino–electron elastic scattering and via CEνNS processes. While solar and other astrophysical neutrinos will ultimately limit the sensitivity to dark matter, they bring forth exciting signatures in their own right. Other compelling searches are those for ultra-rare, second-order weak processes, for these provide unique probes of beyond standard model physics. While the current generation of detectors(LZ, PandaX-4T and XENONnT) continue to acquire data in different deep underground laboratories, next-generation experiments at the multi-ten-ton scale (DARWIN/XLZD and PandaX-xT) are being planned, with large-scale research and development projects and prototyping ongoing. With foreseen first data in the early 2030s, these detectors might discover dark matter particles, and thus solve a problem which is almost a century old. At the same time, they will break new grounds in other areas of astroparticle physics, in particular in neutrino physics and rare nuclear decays. Certainly this will not be the final generation of Xe-TPCs. To make sense of future observations, including those which are unexpected, it is probable that a series of new, larger and more sophisticated experiments will need to be designed. To paraphrase the authors of Aprile *et al.* [6], we believe that the best pages in the history of dual-phase Xe-TPCs are yet to be written.

## Data Availability

This article has no additional data.

## Footnotes

1 Superior energy resolutions can be achieved in high-pressure gas TPCs with electroluminiscent amplification, see [28]. As a concrete example, a relative resolution of 0.4% was obtained in the 5 kg scale NEXT-White detector [29], a prototype for NEXT [30].

2 A ${\;}^{222}\mathrm{Rn}$ concentration of $0.1\;\mu \mathrm{Bq}\;{\mathrm{kg}}^{-1}$ corresponds to less than one radon atom per 100 mol of xenon. The main background is due to ${\;}^{214}\mathrm{Bi}$ β-decays which are not accompanied by an α-decay and thus cannot be tagged in the TPC.

3 Most likely the XENON1T low-energy excess was indeed caused by tritium decays, since in XENONnT the fluid handling and purification is different and measures to reduce any initial tritium concentration were taken.

4 The manuscript was received in October 1973, only a few months after the Gargamelle collaboration had presented the first evidence of the weak neutral current in July of the same year [65].

5 We refer to Xiang *et al.* [68] for a recent study of uncertainties of low-energy LXe responses to nuclear recoils and their effect on solar ${\;}^{8}B$ neutrino searches.
